# Parental burnout and depression among Iranian mothers: The mediating role of Maladaptive Coping modes

**DOI:** 10.1002/brb3.2900

**Published:** 2023-02-27

**Authors:** Narges Sabzi, Zohreh Khosravi, Bessat Kalantar‐Hormozi

**Affiliations:** ^1^ Department of Psychology, Faculty of Education and Psychology Shahid Beheshti University Tehran Iran; ^2^ Department of Psychology, Faculty of Education and Psychology Alzahra University Tehran Iran; ^3^ Department of clinical psychology, Faculty of Psychology and Education Kharazmi University Tehran Iran

**Keywords:** coping modes, COVID‐19, depression, pandemic, parental burnout, structural equation modeling

## Abstract

**Objective:**

Depression in mothers impacts children negatively. Understanding the antecedents and the underlying mechanisms of depression is essential in helping clinicians target depressive symptoms effectively. This study investigated the relationship between parental burnout and depression in mothers and examined the mediation role of Maladaptive Coping modes.

**Method:**

A total of 224 mothers participated in this study and completed the Parental Burnout Assessment scale, Patient Health Questionnaire, and items related to coping modes in Schema Mode Inventory.

**Results:**

Data analysis with structural equation modeling revealed that depression and parental burnout were positively and significantly related. Bootstrap analysis showed that all coping modes except the Self‐Aggrandizer mode act as mediators between parental burnout and depression in mothers. Detached Protector mode had the strongest indirect effect on depression.

**Conclusion:**

The results suggest Maladaptive Coping modes mediate the association between parental burnout and depression. The present finding provides evidence that Maladaptive Coping modes can be considered probable mediational mechanisms that relate depression to parental burnout in mothers and may serve as potential interventional targets.

## Graphical abstract

Dysfunctional coping modes mediate the association between parental burnout and depression in mothers.

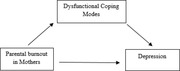

Schools were mainly run online in Iran throughout the Covid‐19 pandemic. Available studies indicate homeschooling during the pandemic has impacted mothers’ mental health unfavorably (Petts et al., 2021; Prados & Zamarro, 2020).

Maternal mental health deserves consideration, not only due to a higher risk of depression and anxiety in women (American Psychiatric Association, 2013), but also because it directly impacts children's development (Wolford et al., 2019; Martins & Gaffan, 2000). Ertel et al. (2011) estimated that 10.2% of mothers suffer from major depressive disorder in any given year. Payab et al. (2012) reported 51.4% of Iranian mothers with primary school–aged children suffer from depressive symptoms.

Kawamoto et al. (2018) suggested parental burnout is an antecedent for both depression and sleep issues (which are related to depression) (Nutt et al., 2008; Kalantar‐Hormozi et al., 2022). Parental burnout is manifested as pronounced exhaustion related to one's parental duties. Burned‐out parents doubt their own ability to be a good parent and feel emotionally detached from their children (Mikolajczak et al., 2019). Potential factors that may help account for the association between parental burnout and depression are not discussed in the literature.

Schema Therapy, first formulated by Jeffrey Young for personality disorders, is now used for a wide range of mental health issues, including depression (Carter et al., 2013; Renner et al., 2016). In recent years, the Schema Therapy is becoming more focused on the Schema mode model (Arntz et al., 2021). Schema modes are momentary states that contain emotional, behavioral, and cognitive components and arise in response to triggering circumstances (Young et al., 2006). These modes can shift but tend to have a persistent nature. The four main categories of Schema modes are as follows: Inner Child modes, Maladaptive Parent modes, Maladaptive Coping modes, and Healthy modes (Young et al., 2006; Arntz & Jacob, 2012) (see Appendix A). Maladaptive Coping modes include Compliant Surrender, Detached Protector, Detached Self‐Soother, Self‐Aggrandizer, and Bully and Attack mode (Young et al, 2006).

The current study proposes that Maladaptive Coping modes can mediate the association between parental burnout and depression. Previous research studies demonstrate that depression and Maladaptive Coping modes are positively related, and avoidant coping strategies are more pervasive in depressed patients (Basile et al., 2018). Nia et al. (2014) reported that overcompensation is the most prevalent Maladaptive Coping mode in depressed Iranian women. Simpson et al. (2018) showed that Detached Protector mode is a significant predictor of emotional exhaustion. Abeltina and Rascevska (2021) suggested Detached Protector and Self‐Aggrandizer modes contribute to burnout pathology.

To our knowledge, the relationship among parental burnout, coping modes, and depression has not yet been investigated and therefore this study investigates if (1) parental burnout is positively related to depression; (2) parental burnout is positively related to Maladaptive Coping modes; (3) Maladaptive Coping modes are positively related to depression; and (4) Maladaptive Coping modes mediate the relationship between parental burnout and depression.

The conceptualized model for this study is illustrated in Figure 1.

**FIGURE 1:**
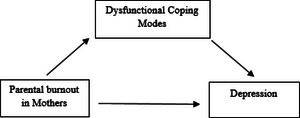
The conceptualized model of the study.

## 1 METHODS

### 1.1 Participants and procedures

This study was a cross‐sectional correlational study. Data collection was performed online. Questionnaires were accessible online through Google forms. Announcements were made via Instagram, Telegram, and WhatsApp. Moreover, messages were sent to colleagues for a further spread of the announcements. Participants accessed the questionnaires after they provided consent to participate. The objectives of the research and inclusion/exclusion criteria were explained to the participants in the note accompanying the form. Inclusion criteria included (1) being a mother with primary school–aged children; (2) willing to complete the research questionnaires voluntarily. Exclusion criteria included (1) having been previously diagnosed with internal or psychological disorders; (2) taking medication that influences sleep duration; and (3) having a child or family member with special needs/ disabilities.

Participants’ characteristics are available in Section 2. Confidentiality was ensured through anonymous completion of the forms. Access to responses was restricted to the research team members for data analysis. Data collection was performed between 24 November 2021 and 24 December 2021.

### 1.2 Measures

#### 1.2.1 Demographic questionnaire

The participants completed information about their educational level, age, marital, and employment status and also the number of their children.

#### 1.2.2 Depression

Patient Health Questionnaire (PHQ‐9) (Kroenke et al. (2001) was used for evaluating depression in the sample. PHQ‐9 is a self‐report measure consisting of nine questions referring to symptoms experienced by the patients during the 2 weeks prior to responding to the questionnaire. Items such as “Little interest or pleasure in doing things” and “Trouble falling asleep, staying asleep, or sleeping too much” are scored from 0 (not at all), 1 (several days), 2 (more than half of the days) to 3 (nearly every day), whereas total score ranges from 0 to 27. The internal reliability and test–retest reliability of PHQ‐9 were reported to be excellent (Cronbach's alpha value .89) in a study by Kroenke et al. (2001) who developed the scale. The psychometric characteristics of Farsi translation of PHQ‐9 were assessed by a study by Farrahi et al. (2021) that reported Cronbach's alpha coefficient of .85 for this questionnaire. In the current study, both Cronbach's alpha coefficient and McDonald's omega coefficient were .90.

#### 1.2.3 Parental burnout

The self‐report Parental Burnout Assessment scale (PBA) created by Roskam et al. (2018) was used to investigate parental burnout among participants. This scale includes 23 items, such as “I feel completely run down by my role as a parent” and “I cannot take being a parent anymore.” Responses are chosen from a 7‐point Likert scale. The scores range from never (0 points) to every day (6 points). PBA and has four subscales: emotional distancing, exhaustion of parental role, feeling of being fed up, and contrast with the parental self in the past. The Persian version of the questionnaire was validated by Mousavi et al. (2020) who reported Cronbach's alpha value between .60 and .93 for the subscales in the Iranian population. Cronbach's alpha value and McDonald's omega coefficient of this measure were both .97 in the present study.

#### 1.2.4 Coping modes

The short SMI (Schema Mode Inventory) is a self‐report tool comprising 14 factors and 118 items. Lobbestael et al. (2010) developed the scale report (*α*) values between .79 and .96 for different modes. The Persian version of this measure was validated by Ghahari et al. (2021). As the conceptualized model of this study only included coping modes, 39 items of the SMI that presented the coping modes were used. The items of the scale are measured using a Likert structure ranging from 1 (never) to 6 (always). In the present study, Cronbach's alpha values for the Compliant Surrender, Detached Protector, Self‐Soother, Self‐Aggrandizer, and Bully and Attack modes were, respectively, .85, .88, .77, .76, and .76, and McDonald's omega coefficients for the mentioned modes were .85, .89, .78, .76, and .77.

### 1.3 Statistical analysis

The collected data (which did not allow for missing data due to online survey) was analyzed using SPSS 26 (for descriptive analysis). Structural equation modeling (SEM) was employed for statistical analysis using AMOS 24 software. Depression, parental burnout, and coping modes were assigned as the endogenous variable, the exogenous variable, and mediator variables, respectively. Mediation analysis was performed using the bootstrap method in AMOS. The acceptable level of error was (*p* < .05).

## 2 RESULTS

### 2.1 Demographic characteristics of the sample

A total of 224 mothers took part in this study (*n* = 224) who were between 25 and 51‐year old with a mean age of 36.86 (SD = 5.08). A total of 209 participants (93.3%) were married, 12 participants (5.4%) were divorced, and 3 participants (1.3%) were widowed. Overall, 65.2% (*n* = 146) were homemaker, and 34.8% (*n* = 78) were employed. A total of 39 participants (17.4%) had graduate degrees, 49.1% (*n* = 110) reported having a bachelor's degree, and 33.5% (*n* = 75) had no university degree. Overall, 31.3% (*n* = 70) were mothers with one child, 52.2% (*n* = 117) had two children, and 16.4% (*n* = 37) had three or more children.

### 2.2 Descriptive statistics

Correlations were calculated using the Pearson coefficient in SPSS (see Table 1). Skewness and kurtosis values were between −2 and +2; therefore, data distribution was normal. Multivariate normality was checked using Mardia's coefficient and the Mahalanobis distance in AMOS, and outliers were corrected according to the obtained values. Table 1 reports descriptive statistics for research variables, including mean and standard deviation for each variable. Table 2 presents the partial correlations among the variables.

**TABLE 1:** Variable descriptive analysis and correlations

Variables	Mean	SD	*S*	*K*	Parental burnout	Compliant Surrender	Detached Protector	Self‐Soother	Self‐Aggrandizer	Bully and Attack	Depression
Parental burnout	30.86	35.29	1.53	1.38	.79						
Compliant Surrender	11.13	4.73	1.09	1.28	^***^.43	.73					
Detached Protector	16.81	7.09	1.34	1.97	^***^.57	^***^.52	.68				
Self‐Soother	10.38	3.40	0.38	−0.08	^***^.28	^***^.24	^***^.25	.75			
Self‐Aggrandizer	22.12	6.53	0.85	1.66	^***^.26	^***^.26	^***^.34	^**^.22	.52		
Bully and Attack	19.70	6.63	0.54	0.22	^***^.31	^***^.35	^***^.37	^***^.24	^***^.45	.56	
Depression	7.83	6.31	0.94	0.26	^***^.64	^***^.44	^***^.57	^***^.31	^***^.24	^**^.22	.71

*Note*: The square root of the average variance extracted (AVE) is reported in the diagonal.

**p ≤ .01, ***p ≤ .001.

**TABLE 2:** Partial correlations between study variables

Coping modes	Parental burnout				Depression			
	**Zero‐order**		**Controlled for depression**		**Zero‐order**		**Controlled for parental burnout**	
	*r*	*p*	*r*	*p*	*r*	*p*	*r*	*p*
Compliant Surrender	.43	<.001	.22	.001	.44	<.001	.24	<.001
Detached Protector	.57	<.001	.33	<.001	.57	<.001	.32	<.001
Self‐Soother	.28	<.001	.11	.089	.31	<.001	.18	.009
Self‐Aggrandizer	.26	<.001	.14	.032	.24	<.001	.10	.136
Bully and Attack	.31	<.001	.23	.001	.22	<.001	.03	.640

As indicated in the previous tables, depression is positively correlated with all the coping modes and also with parental burnout (*p* < .05). Depression had the strongest correlation with parental burnout (.64) and Detached Protector mode (.57), respectively. Parental burnout and all coping modes demonstrated positive correlation (*p* < .05). Parental burnout had the strongest correlation with Detached Protector mode (.57) and Compliant Surrender mode (.43), respectively. The discriminant validity was assessed using the Fornell and Larcker (1981) criteria by comparing the square root of the average variance extracted (AVE) in the diagonal with the correlation coefficients (off‐diagonal) for each construct in the relevant rows and columns. The square roots of the AVE are underlined in Table 1. All AVE values are greater than the correlations of that variable with other variables; therefore, discriminate validity is confirmed for all variables.

### 2.3 Measurement model

The measurement model was tested using AMOS. Factor loadings were checked in confirmatory factor analysis, and items 1, 7, 17, 26, and 39 of the coping mode variables were eliminated from the model because the values were less than .4. The validity and reliability of the research questionnaires are reported in Table 3. The reliability values are all higher than .70. Validity values were all acceptable. A summary of reliability and validity values of the research variables is presented in Table 3.

**TABLE 3:** Reliability and validity of variables

AVE	Composite reliability	Cronbach's alpha	Variables
.62	.98	.97	Parental burnout
.54	.88	.85	Compliant Surrender
.46	.89	.88	Detached Protector
.56	.81	.77	Self‐Soother
.27	.80	.76	Self‐Aggrandizer
.31	.82	.76	Bully and Attack
.50	.93	.90	Depression

Abbreviation: AVE, average variance extracted.

### 2.4 Structural model

**FIGURE 2:**
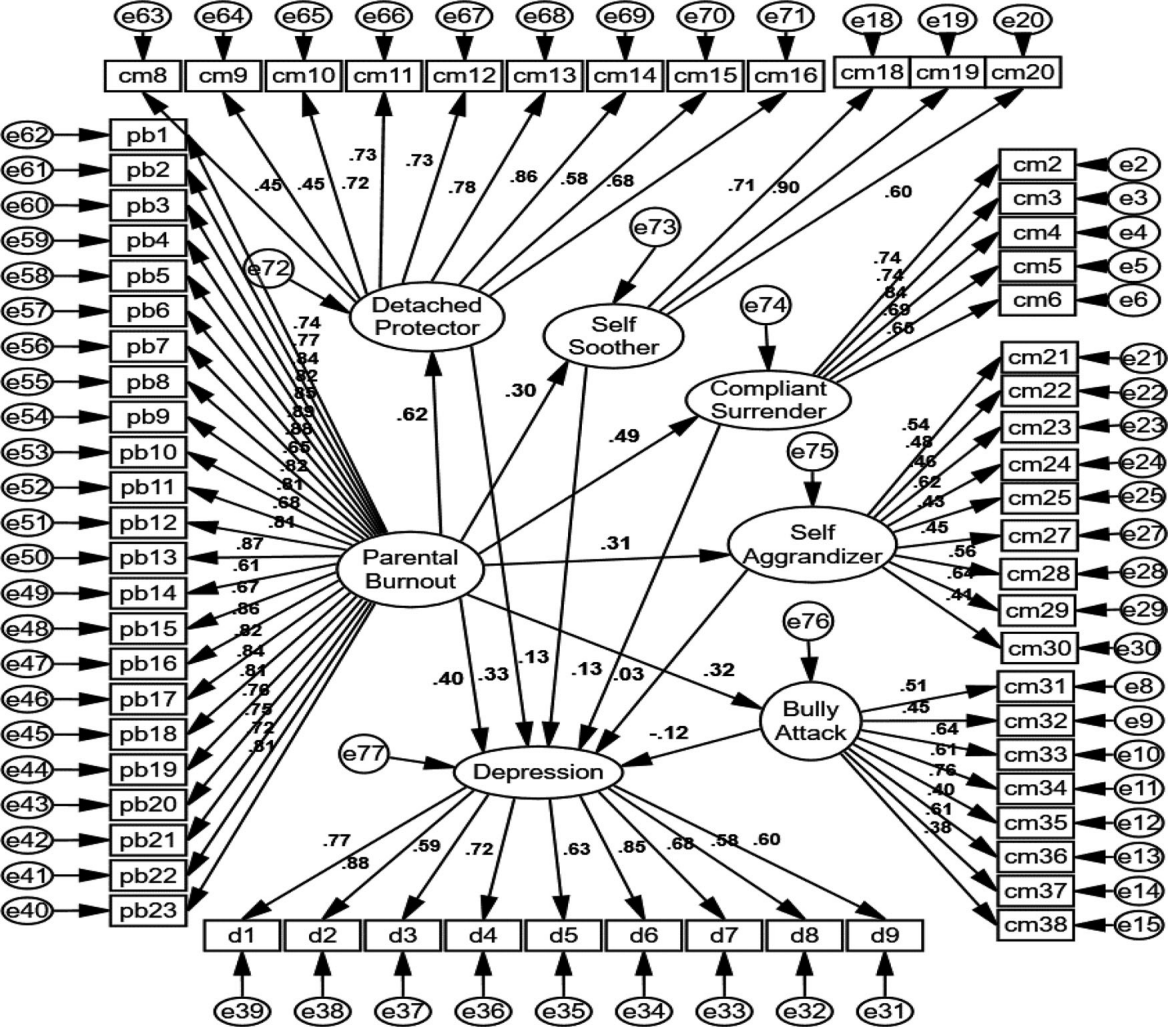
Measurement model in standardized mode.

Figure 2 presents the depicted model in AMOS in standardized mode. Adjusted goodness of fit index (AGFI), parsimonious goodness of fit index (PGFI), incremental fit index (IFI), normed fit index (NFI), comparative fit index (CFI), goodness of fit index (GFI), root mean square error of approximation (RMSEA), and normed chi‐square (chi square/df) were used to assess the fitness of the model. Table 4 shows the model has an acceptable fit. The coefficient of determination for depression was .57, which indicates parental burnout and coping modes explained 57% of the variance for depression.

**TABLE 4:** Model fit summary

*R* ^2^	AGFI	PGFI	IFI	NFI	CFI	GFI	RMSEA	Chi square/df	Fit index
.33<	.70<	.70<	.90<	.90<	.90<	.90<	.08>	Between 1 and 5	Criteria
.57	.79	.76	.94	.89	.93	.92	.07	2.26	Result

Abbreviations: AGFI, adjusted goodness of fit index; CFI, comparative fit index; GFI, goodness of fit index; IFI, incremental fit index; NFI, normed fit index; PGFI, parsimonious goodness of fit index; RMSEA, root mean square error of approximation.

Table 5 shows that 10 out of 11 direct paths were significant (*p* < .05), and the only insignificant path was Self‐Aggrandizer and depression path (*p* > .05). The path between the Bully and Attack mode and depression was narrowly confirmed because *t* value was 1.96, and significance level was .05. Results show parental burnout was correlated with depression and all five coping modes. Mediation analysis was performed using the bootstrap method.

**TABLE 5:** Standard regression weights for the direct model

*p*	*t*	SE	Standardized coefficient	Unstandardized coefficient	Direct paths
<.001	3.33	.678	.49	2.26	Parental burnout → Compliant Surrender
.001	3.23	.528	.62	1.70	Parental burnout → Detached Protector
.006	2.74	.502	.30	1.37	Parental burnout → Self‐Soother
.005	2.81	.356	.31	1.00	Parental burnout → Self‐Aggrandizer
.007	2.72	.484	.32	1.31	Parental burnout → Bully and Attack
.004	2.91	.315	.40	0.92	Parental burnout → depression
.035	2.10	.032	.13	0.07	Compliant Surrender → depression
<.001	3.78	.074	.33	0.28	Detached Protector → depression
.027	2.21	.029	.13	0.06	Self‐Soother → depression
.656	.45	.043	.03	0.02	Self‐Aggrandizer → depression
.050	1.96	.034	.12	0.07	Bully and Attack → depression

The results from Table 6 indicate that all coping modes except the Self‐Aggrandizer mode mediate the relationship between parental burnout and depression (*p* < .05), and the Detached Protector mode was the strongest mediator with an indirect effect equal to .20.

**TABLE 6:** Bootstrap estimates and standard errors

***p***	**SE**	**Indirect model**	**Paths**
2.56	.025	.064	Parental burnout → Compliant Surrender → depression
4.27	.048	.205	Parental burnout → Detached Protector → depression
2.29	.017	.039	Parental burnout → Self‐Soother → depression
.64	.014	.009	Parental burnout → Self‐Aggrandizer → depression
2.11	.018	.038	Parental burnout → Bully and Attack → depression

An alternative model evaluating the mediating role of parental burnout in the association between coping modes and depression was additionally tested, which was not identified by AMOS. Therefore, the results of this alternative model are not reported but are present in Appendix B.

## 3 DISCUSSION

Findings of this study provide preliminary support for the role of Maladaptive Coping modes in explaining the association between parental burnout and depression in mothers of primary school–aged children. Overall, the proposed model was a good fit for the data. This finding is consistent with Schema theory that suggests distressing life events or circumstances are dealt with by shifting to certain Schema modes, and these Schema modes are related to psychopathology (Young et al., 2006).

The results of the current study, in‐line with literature (Kawamoto et al., 2018), confirm that parental burnout and depression are related (confirmation of the first hypothesis). This finding was predictable based on previous studies which explain that even though depression and burnout are different constructs, they have many similarities and share the same risk factors (Mikolajczak et al., 2020; Schonfeld et al., 2018).

Parental burnout was associated with all five Maladaptive Coping modes (confirmation of the second hypothesis). To our knowledge, the mentioned association has not been investigated before, so this is a new finding. We can explain this by reflecting on the elements of parental burnout and the Schema theory. Central to the parental burnout construct is emotional exhaustion and detachment that are the result of parental roles. These parental roles and the accompanying stress can act as environmental and psychological triggers for shifting between different states of mind and perhaps making mothers more prone to making use of Maladaptive Coping modes.

Results show most Maladaptive Coping modes are related to depression and play a mediational role in the relationship between parental burnout and depression (confirmation of the third and fourth hypotheses). It was demonstrated that the Self‐Aggrandizer mode was the only mode that was not correlated to depression and did not mediate the relationship between parental burnout and depression. This finding is consistent with previous studies in which self‐aggrandizing or grandiosity was related to depression only when narcissistic personality traits or bipolar disorder are present (Kealy et al., 2012; Fjermestad‐Noll et al., 2019; Hawke & Provencher, 2012; Picardi et al., 2018). The Detached Protector mode was shown to be the strongest mediator linking parental burnout to depression. This finding aligned with the study performed by Basile et al. (2018) who also found a strong correlation between this mode and depression. The Detached Protector mode is characterized by withdrawal and supersession of emotions. Available research has already confirmed that emotional suppression and depressive symptoms are related with each other (Beblo et al., 2012; Gross & John, 2003; John & Gross, 2004).

The global pandemic has affected families and mothers across the world and has urged psychologists to investigate appropriate psychotherapeutic interventions that can help mothers manage the consequences of the pandemic. This study contributes to our knowledge of underlying mechanisms that facilitate the effect of parental burnout on depression. According to the results of this study, clinicians may benefit from focusing on Schema modes while working with mothers who suffer from depressive symptoms and exhaustion originating from parental burnout during the pandemic. Moreover, the results of this research also add to previous findings available on the usefulness of Schema Therapy for depression.

### 3.1 Limitations and implications for further research

Some limitations need to be highlighted. This study was based on the cross‐sectional design, which limits causal inference. To identify the causal relationships, further longitudinal and experimental studies are needed. Another limitation is that the study findings are based on the responses from self‐administered online questionnaires that may be influenced by subjective bias and also participant profile bias. Structured interview was not available.

Our hypotheses were based on our assumptions and understanding of the current research literature. Thus, other researchers may consider different indirect and direct effects. Moreover, based on cultural issues in Iran, we assumed mothers as the primary caregivers involved in homeschooling, and therefore, we only included mothers in this research. The inclusion of fathers is recommended in similar future studies. The sample size was sufficient for performing structural equation modeling (Kline, 2015), but we recommend a larger sample in future studies to further confirm the results. Despite these limitations, the current study provides a framework for the researchers through testing the mediating effects of coping modes in the relationship between parental burnout and depression in mothers.

## 4 CONCLUSION

This study investigated the relationship between parental burnout, depression, and Maladaptive Coping modes. The results suggest parental burnout is positively correlated with both depression and Maladaptive Coping modes. Moreover, Maladaptive Coping modes mediate this association. The present findings provide further evidence that Maladaptive Coping modes can be considered possible mediational mechanisms in psychopathology of depression and may serve as important targets during psychological interventions.

## CONFLICT OF INTEREST STATEMENT

The authors declare no conflict of interests.

## Data Availability

The data analyzed for the results of this research is available from the corresponding author on reasonable request.

## APPENDIX A

### A.1

A summary of Schema modes.

**Table:**

Innate Child modes	Angry child	feels infuriated, frustrated, needy, or impatient because her core emotional (or physical) needs have not been met
	Vulnerable child	feels empty, alone, socially unacceptable, and undeserving of love, because his most important emotional needs have not been met
	Enraged child	experiences feelings of anger, hurts or damages people or objects, anger is out of control mostly when his autonomy is threatened
	Impulsive child	acts on her desires or impulses selfishly, cannot tolerate limits, and appears spoiled
	Undisciplined child	cannot force himself to finish routine or boring tasks, gets frustrated quickly and gives up
	Happy child	at peace because her core emotional needs have been met, feels loved, safe, and competent
Inner Critic modes (formerly known as parent modes)	Punitive Critic	the internalized voice of caretakers criticizing and punishing the person, becomes angry at herself and feel that she deserves punishment for having or showing normal needs. Harsh, critical, unforgiving, loathes oneself, and/or others
	Demanding Critic	continually pushes and puts pressure on oneself and others to meet excessively high standards
Maladaptive Coping modes	Compliant Surrender	acts in a submissive, reassurance‐seeking, or self‐deprecating way toward others out of fear of conflict or rejection
	Detached Self‐Soother	tunes down negative emotions by engaging in activities, for example, workaholism or taking substances that will somehow soothe, stimulate, or distract him
	Detached Protector	shuts down emotions, disconnects from others, rejects help, appears emotionally distant, and avoids getting close to people, emptiness, boredom, and psychosomatic complaints
	Self‐Aggrandizer	feels superior, special, and powerful and entitled, competitive, grandiose, abusive, concerned about appearances rather than feelings, little compassion for others, does not want to follow the rules that apply to everyone else
	Bully/Attack	uses threats, intimidation, aggression, and coercion to get what he wants, retaliation, directly harms others in a controlled way, be it emotionally, physically, sexually, verbally, or through antisocial or criminal acts
Integrative mode	Healthy adult	adaptive validating and self‐regulating aspect of a person, functional problem solving, responsible, and committed

*Source*: Adapted with permission from Roediger et al. (2018).

## APPENDIX B

### B.1

Alternative model: regression weights for the second model

**Table:**

			Estimate	SE	CR	*p*	Label
Burnout	←	Bully	.039				
Burnout	←	Self‐Aggrandizer	.127				
Burnout	←	Detached	1.475				
Burnout	←	Compliant	.358				
Burnout	←	Soother	.208				
Depression	←	Compliant	.069	.029	2.401	.016	
Depression	←	Self‐Aggrandizer	.023	.042	.532	.595	
Depression	←	Burnout	.110				
Depression	←	Bully	−.062	.031	‐2.000	.045	
Depression	←	Soother	.064	.028	2.292	.022	
Depression	←	Detached	.282	.071	3.951	***	
cm2	←	Compliant	1.000				
cm3	←	Compliant	.923	.089	10.409	***	
cm4	←	Compliant	.971	.085	11.480	***	
cm5	←	Compliant	1.017	.105	9.727	***	
cm6	←	Compliant	.812	.090	9.040	***	
cm31	←	Bully	1.000				
cm32	←	Bully	.933	.181	5.165	***	
cm33	←	Bully	.972	.148	6.557	***	
cm34	←	Bully	.900	.144	6.262	***	
cm35	←	Bully	1.250	.180	6.961	***	
cm36	←	Bully	.587	.135	4.356	***	
cm37	←	Bully	.832	.134	6.222	***	
cm38	←	Bully	.244	.053	4.632	***	
cm18	←	Soother	1.000				
cm19	←	Soother	1.334	.159	8.383	***	
cm20	←	Soother	.934	.115	8.136	***	
cm21	←	Self‐Aggrandizer	1.000				
cm22	←	Self‐Aggrandizer	.985	.195	5.046	***	
cm23	←	Self‐Aggrandizer	1.080	.210	5.148	***	
cm24	←	Self‐Aggrandizer	1.190	.197	6.050	***	
cm25	←	Self‐Aggrandizer	.767	.156	4.918	***	
cm27	←	Self‐Aggrandizer	.807	.159	5.077	***	
cm28	←	Self‐Aggrandizer	1.327	.231	5.735	***	
cm29	←	Self‐Aggrandizer	1.074	.175	6.147	***	
cm30	←	Self‐Aggrandizer	.962	.205	4.689	***	
d9	←	Depression	1.000				
d8	←	Depression	1.008	.144	6.976	***	
d7	←	Depression	1.481	.189	7.826	***	
d6	←	Depression	1.784	.196	9.086	***	
d5	←	Depression	1.342	.181	7.397	***	
d4	←	Depression	1.576	.193	8.178	***	
d3	←	Depression	1.270	.181	7.031	***	
d2	←	Depression	1.816	.197	9.238	***	
d1	←	Depression	1.539	.181	8.519	***	
pb23	←	Burnout	.893				
pb22	←	Burnout	.882				
pb21	←	Burnout	.852				
pb20	←	Burnout	.841				
pb19	←	Burnout	.841				
pb18	←	Burnout	.919				
pb17	←	Burnout	.940				
pb16	←	Burnout	.834				
pb15	←	Burnout	.904				
pb14	←	Burnout	.812				
pb13	←	Burnout	.932				
pb12	←	Burnout	.726				
pb11	←	Burnout	.670				
pb10	←	Burnout	1.029				
pb9	←	Burnout	1.014				
pb8	←	Burnout	.880				
pb7	←	Burnout	.892				
pb6	←	Burnout	.949				
pb5	←	Burnout	.988				
pb4	←	Burnout	.995				
pb3	←	Burnout	1.037				
pb2	←	Burnout	.831				
pb1	←	Burnout	.832				
cm8	←	Detached	1.000				
cm9	←	Detached	.588	.117	5.035	***	
cm10	←	Detached	1.263	.197	6.426	***	
cm11	←	Detached	1.658	.257	6.458	***	
cm12	←	Detached	1.609	.250	6.445	***	
cm13	←	Detached	1.714	.258	6.648	***	
cm14	←	Detached	1.929	.282	6.848	***	
cm15	←	Detached	1.169	.201	5.829	***	
cm16	←	Detached	1.176	.187	6.289	***	

*** 0.001.

Standardized regression weights for the second model

**Table:**

			Estimate
Burnout	←	Bully	.021
Burnout	←	Self‐Aggrandizer	.050
Burnout	←	Detached	.514
Burnout	←	Compliant	.210
Burnout	←	Soother	.122
Depression	←	Compliant	.146
Depression	←	Self‐Aggrandizer	.032
Depression	←	Burnout	.396
Depression	←	Bully	‐.122
Depression	←	Soother	.135
Depression	←	Detached	.351
cm2	←	Compliant	.734
cm3	←	Compliant	.746
cm4	←	Compliant	.837
cm5	←	Compliant	.696
cm6	←	Compliant	.646
cm31	←	Bully	.523
cm32	←	Bully	.452
cm33	←	Bully	.660
cm34	←	Bully	.607
cm35	←	Bully	.755
cm36	←	Bully	.362
cm37	←	Bully	.600
cm38	←	Bully	.391
cm18	←	Soother	.704
cm19	←	Soother	.918
cm20	←	Soother	.595
cm21	←	Self‐Aggrandizer	.515
cm22	←	Self‐Aggrandizer	.461
cm23	←	Self‐Aggrandizer	.475
cm24	←	Self‐Aggrandizer	.622
cm25	←	Self‐Aggrandizer	.444
cm27	←	Self‐Aggrandizer	.465
cm28	←	Self‐Aggrandizer	.564
cm29	←	Self‐Aggrandizer	.642
cm30	←	Self‐Aggrandizer	.416
d9	←	Depression	.576
d8	←	Depression	.562
d7	←	Depression	.660
d6	←	Depression	.837
d5	←	Depression	.609
d4	←	Depression	.704
d3	←	Depression	.568
d2	←	Depression	.862
d1	←	Depression	.751
pb23	←	Burnout	.787
pb22	←	Burnout	.694
pb21	←	Burnout	.719
pb20	←	Burnout	.736
pb19	←	Burnout	.784
pb18	←	Burnout	.821
pb17	←	Burnout	.805
pb16	←	Burnout	.845
pb15	←	Burnout	.641
pb14	←	Burnout	.585
pb13	←	Burnout	.856
pb12	←	Burnout	.791
pb11	←	Burnout	.656
pb10	←	Burnout	.782
pb9	←	Burnout	.798
pb8	←	Burnout	.623
pb7	←	Burnout	.860
pb6	←	Burnout	.874
pb5	←	Burnout	.833
pb4	←	Burnout	.804
pb3	←	Burnout	.824
pb2	←	Burnout	.748
pb1	←	Burnout	.710
cm8	←	Detached	.450
cm9	←	Detached	.445
cm10	←	Detached	.718
cm11	←	Detached	.727
cm12	←	Detached	.723
cm13	←	Detached	.787
cm14	←	Detached	.864
cm15	←	Detached	.577
cm16	←	Detached	.681

*Model fit summery for the second tested model*

**CMIN**

**Table:**

Model	NPAR	CMIN	df	*p*	CMIN/df
Default model	144	4752.918	2068	.000	2.298
Saturated model	2211	.000	0		
Independence model	66	12484.131	2145	.000	5.820

**RMR, GFI**

**Table:**

Model	RMR	GFI	AGFI	PGFI
Default model	.307	.582	.553	.544
Saturated model	.000	1.000		
Independence model	.907	.122	.095	.118

**Baseline comparisons**

**Table:**

Model	NFI Delta1	RFI rho1	IFI Delta2	TLI rho2	CFI
Default model	.619	.605	.742	.731	.740
Saturated model	1.000		1.000		1.000
Independence model	.000	.000	.000	.000	.000

**Parsimony‐adjusted measures**

**Table:**

Model	PRATIO	PNFI	PCFI
Default model	.964	.597	.714
Saturated model	.000	.000	.000
Independence model	1.000	.000	.000

**NCP**

**Table:**

Model	NCP	LO 90	HI 90
Default model	2684.918	2488.033	2889.400
Saturated model	.000	.000	.000
Independence model	10339.131	9991.047	10693.879

**FMIN**

**Table:**

Model	FMIN	F0	LO 90	HI 90
Default model	21.314	12.040	11.157	12.957
Saturated model	.000	.000	.000	.000
Independence model	55.983	46.364	44.803	47.955

**RMSEA**

**Table:**

Model	RMSEA	LO 90	HI 90	PCLOSE
Default model	.076	.073	.079	.000
Independence model	.147	.145	.150	.000

**AIC**

**Table:**

Model	AIC	BCC	BIC	CAIC
Default model	5040.918	5164.610	5532.195	5676.195
Saturated model	4422.000	6321.192	11965.149	14176.149
Independence model	12616.131	12672.823	12841.299	12907.299

**ECVI**

**Table:**

Model	ECVI	LO 90	HI 90	MECVI
Default model	22.605	21.722	23.522	23.160
Saturated model	19.830	19.830	19.830	28.346
Independence model	56.575	55.014	58.165	56.829

**HOELTER**

**Table:**

Model	HOELTER .05	HOELTER .01
Default model	103	105
Independence model	41	42

**Execution time summary**

**Table:**

Minimization	.086
	.086
Miscellaneous	2.059
Bootstrap	.000
Total	2.145
